# A Distributed Multiagent System Architecture for Body Area Networks Applied to Healthcare Monitoring

**DOI:** 10.1155/2015/192454

**Published:** 2015-03-22

**Authors:** Filipe Felisberto, Rosalía Laza, Florentino Fdez-Riverola, António Pereira

**Affiliations:** ^1^Fundação Para a Ciência e a Tecnologia (FCT), Foundation for Science and Technology, 1249-074 Lisbon, Portugal; ^2^Higher Technical School of Computer Engineering, University of Vigo, Polytechnic Building, Campus Universitario As Lagoas s/n, 32004 Ourense, Spain; ^3^INOV INESC INNOVATION, Institute of New Technologies of Leiria, 2411-901 Leiria, Portugal; ^4^Computer Science and Communications Research Centre, School of Technology and Management, Polytechnic Institute of Leiria, 2411-901 Leiria, Portugal

## Abstract

In the last years the area of health monitoring has grown significantly, attracting the attention of both academia and commercial sectors. At the same time, the availability of new biomedical sensors and suitable network protocols has led to the appearance of a new generation of wireless sensor networks, the so-called wireless body area networks. Nowadays, these networks are routinely used for continuous monitoring of vital parameters, movement, and the surrounding environment of people, but the large volume of data generated in different locations represents a major obstacle for the appropriate design, development, and deployment of more elaborated intelligent systems. In this context, we present an open and distributed architecture based on a multiagent system for recognizing human movements, identifying human postures, and detecting harmful activities. The proposed system evolved from a single node for fall detection to a multisensor hardware solution capable of identifying unhampered falls and analyzing the users' movement. The experiments carried out contemplate two different scenarios and demonstrate the accuracy of our proposal as a real distributed movement monitoring and accident detection system. Moreover, we also characterize its performance, enabling future analyses and comparisons with similar approaches.

## 1. Introduction

The still prevailing world economic crisis heightened various social problems; some of the more alarming are the ones relating to the aging of most developed countries [1]. The reduction in the active force means there is not only less available income to support the elderly but there are also fewer people to provide continued care. Instead of decreasing this problem, it is expected to grow, and by the year 2050 the European population over 65 will have grown from the current 17.1% to 30.0% [2]. This leads to the necessity of finding an economically viable and dignified solution to support the growing elderly population.

In this context, one area which has gained lots of attention from academia and commercial sectors is health monitoring. With the appearance of biomedical sensors and suitable network protocols, a new generation of wireless sensor networks has emerged: body area networks (BAN). These networks can be used for continuous monitoring of vital parameters, movement, and the surrounding environment. The data gathered by these networks contributes to improve users' quality of life and allows creating useful knowledge bases.

Related with the scope of this study, the work being done at the Center for Future Health of the Medical Center of University of Rochester [3] is aimed at answering an important question. “What can be learned about an individual's health state by observing the motion, activity and interactions in one's natural environment?” The objective of the research is to learn what is normal for the person and to detect and monitor trends that may indicate developmental or incipient health issues and hence detect such conditions in the earliest possible stage.

In this respect, enabling remote monitoring of patients offers potential advantages like reducing internment and aftercare costs, as well as providing speedier delivery of preventive and emergency care, with little to no attention required from the patient. The patient quality of life can also be improved by avoiding confinement to the proximity of nonportable medical monitoring equipment. As a consequence, one interesting application area of BANs to health monitoring is the continuous supervision of elder movements, both during activities of their daily living (ADL) or while being subjected to a specific rehabilitation process.

In the particular case of ADL monitoring, an important field of research is the development of effective solutions able to recognize deterioration in movement, help in rehabilitation, and detect accidents, being the identification of falls a key problem to cope with. Regarding motion and ADL monitoring, the application of BAN technology is prone to errors natural to internal monitoring systems. These types of errors can only be mitigated using complex filtering algorithms or very sensitive sensors. In the early 1990s, Lord and Colvin started studying the acceleration sustained by the human body during an impact using a small triaxial analog accelerometer [4]. This approach was the one followed by the majority of latter studies. After a few years (in 1998) the first prototype of a fall detection device to be used on a telecare system was developed [5].

These first systems had a sub 90% success rate, suffering from many false positives. This situation was motivated in part by the inefficient hardware being used and the way the problem was being approached. As the system relied on the energy of the impact to be transmitted through the human body, the fall could not be detected (or would be confused with ADL) in case the energy was absorbed by the body. In 2008, Bourke et al. introduced a very precise solution for distinguishing falls from ADLs using BANs [6]. Their work was supported by the research of Wu [7] and their own previous work [8] which, using image processing equipment, proved that it was possible to distinguish a fall from an ADL using the velocity of the torso instead of the force of the impact. Nowadays, the introduction of more precise sensors accompanied by powerful and efficient processors has enabled recent research to advance from distinguishing falls from ADLs to actually monitoring said ADLs [9].

However, in this area there is still the need of open architectures that facilitate the design and implementation of sensor network applications, being able to reduce the overall message communication and to optimize energy consumption [10]. In this context, distributed agent-based architectures provide more flexible ways to move functions to where actions are needed, thus obtaining better responses at execution time, autonomy, service continuity, and superior levels of flexibility and scalability than centralized approaches. Moreover, sensors' capabilities can be enhanced by means of intelligent agents, changing dynamically their behavior and personalizing their reactions [11]. A multiagent system possesses mobile, goal oriented, communicative, and reactive agents. These characteristics make this approach suitable for the development of BAN healthcare monitoring applications, in which agents are mainly advantageous given their ability to change according to the nature of surrounding.

The solution presented in this work represents the culmination of a study which started in 2010. The original project evolved from a single node for fall detection [12] into a multisensor solution capable of identifying unhampered falls and analyzing the users' movement [13]. Although the results were promising, the architecture of the previous system did not involve the use of an adaptive model with learning ability and did not guarantee an efficient analysis of the information generated. For this reason, in this work we propose a BAN based on a BDI (belief, desire, and intention) multiagent system (MAS) with the goal of being more robust, flexible, and adaptable. Our multiagent system allows the definition of an open architecture, which is straightforward scalable by easily adding new agents (with the same or different goals) that follow the proposed BDI specification. The advantage of information sharing between agents allows them to correct errors by their ability to make an explicit coordination. Each agent is considered an autonomous software process, which is able to manage its own environmental information and local state based on the global information. The BDI model enables to view agents as goal-directed entities that act in a rational manner.

While this section has introduced and established the basis of the work, the rest of the paper is structured as follows: Section 2 describes related studies in the area of wireless sensor network (WSN), BAN, and MAS applied to healthcare projects.  Section 3 introduces the physical components comprising our underlying system used to perform movement recognition. While Section 4 explains the proposed multiagent system architecture, Section 5 gives details about its implementation and real deployment.  Section 6 introduces the experimental evaluation carried out and discusses the obtained results. Finally, Section 7 draws the conclusions and highlights future work.

## 2. Related Work

Thanks to the significant advances in MEMS (microelectromechanical systems) and CMOS (complementary metal-oxide-semiconductor), WSN technology has come a long way since it was first used in 1967 project Igloo White [14], with the purpose of deploying a wireless network of seismic and acoustic sensors in the Ho Chi Minh trail during the Vietnam War.

With the emergence of WSNs and the rapid growth of physiological sensor technology, scientists from various fields sought to adapt sensor networks for the purposes of health monitoring applications. In 2006, Yang and Yacoub coined the term body sensor network (BSN) to refer to that particular application of WSNs [15]. BAN technology is a natural refinement of that concept [16], providing more flexibility for a broader set of applications but with special attention to the medical sector. BANs, as the name suggests, are networks of computing entities that have the distinctive characteristic of being physically linked to a user's close proximity.

One of the most referenced projects in the area of BAN is Code Blue [17]. It employs ad hoc networks of off-the-shelf motes and medical sensors (e.g., electrocardiogram, peripheral capillary oxygen saturation, electromyogram, and motion) in order to give a response in prehospital and in-hospital emergency care, disaster response triage process, and stroke patient rehabilitation. The same authors of Code Blue were also involved in a related project called Mercury [18], which aims to support high-resolution motion studies of patients with Parkinson's disease, stroke, and epilepsy.

Two relevant projects related to the residential healthcare monitoring component of our system are ALARM-NET [19] and BASUMA [20]. On the one hand, ALARM-NET implements a home healthcare system that integrates environmental and physiological sensors in a scalable and heterogeneous architecture. This development includes an analysis program called Circadian Activity Rhythm (CAR), which processes sensor data for learning individual behavior patterns. On the other hand, the BASUMA project seeks continuous health monitoring of chronically ill patients in their own homes in order to detect when the health state changes to worse. It can alert and recommend actions in a timely manner before critical conditions occur.

Regarding the combined use of MAS and BANs for the development of healthcare monitoring applications, Vaidehi et al. [21] proposed a health care monitoring system based on WSN which is capable of collecting, retrieving, storing, and analyzing vital signs of the patient. The proposed MAS consists of four agents (i.e., admin, control, query, and data agents) which performs data reduction using Epsilon approximation. The use of the data agent reduces data traffic and the requirement for secondary storage space.

From another perspective, the Confidence project [22] applies a multiagent system to correctly distinguish normal from abnormal activities. In this case, the agents are all external to the BAN and the surrounding hardware relies on a combination of sensors placed in the user and sensors placed in the user's home.

Complementarily, Castanedo et al. [23] describe a MAS based on the BDI model for processing information and fusing data coming from a distributed visual sensor network. Their work is more focused on how to fuse a set of tracks which belong to the same object from different agents. This information was used to fuse only data which provides an accurate monitorization, discarding those visual sensors presenting tracking errors.

As stated in Introduction, the detection of abnormal movements seems to be conveniently addressed (i.e., there are methods and algorithms able to effectively process raw data) but, as evidenced in the related work carried out, there is still a substantial room for improvement in the design of open architectures using MAS able to cope with inherent issues regarding the deployment of distributed applications (e.g., tracking, data fusion, learning, etc.). In this context, our proposal brings together both fields: an underlying physical system for efficiently capturing raw data (presented in Section 3) and a multiagent implementation for real deployment (detailed in Sections 4 and 5).

## 3. Underlying Physical System Architecture

One of the biggest struggles in WSNs has always been how to balance between processing power and energy conservation. This problem is even more emphasized in BANs, where usability is another constraint to balance. When devising a BAN it is mandatory to carefully take into account this triangle, or else there is the risk that the system does not fulfill its objectives or it fails to be accepted by final users.

The first two constraints are easy to phantom as both have conditions for their acceptance that can be easily defined: a given BAN is deemed acceptable if (i) it is capable of processing the required amount of information in the specified time and (ii) it is capable of repeating the process during the defined time period. The third constraint is more dependent on subjectivity, but the size of the node, the number of nodes, and their placement are a good common ground. To be able to balance all three of these constraints some concessions had to be made, as the technology available would not allow all of them to be fully satisfied, at least not in an economical viable way.

If the concession was made in terms of usability or energy efficiently, it would mean the users would be constrained in terms of space, movement, autonomy, or even all of the three. Therefore, these types of adjustments will not be discussed as their costs would violate the objectives of the proposed solution. For this reason, our architecture was defined taking into account the necessary limitations in terms of in-BAN processing and, at the same time, taking into consideration the recent advances done in the field of mobile processors, which enable the use of more advanced and intelligent algorithms.

**Figure 1:**
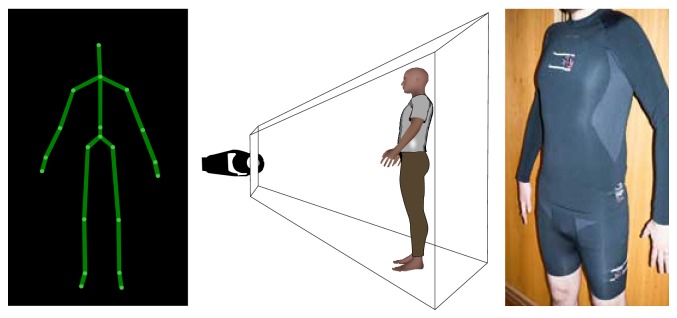
External process for calibrating the nodes used in the implemented BAN.

In particular, this project has strict rules of using noninvasive methods to monitor the user, so in no way could the sensors be physically attached to the human body. This requirement introduces a variable related to the small differences in the node position every time the user puts on the nodes. In order to be possible to precisely evaluate data between usages and specially to be able to viably compare data between users, it was mandatory to introduce an external system capable of correctly evaluating the body part position against its own known referential. As the external system is only necessary during the initial setup, it does not introduce constraints in terms of user's spatial movement or to the user's privacy. For the current version of the architecture the external module used to setup the whole system was the Microsoft Kinect SDK (see Figure 1).

**Figure 2:**
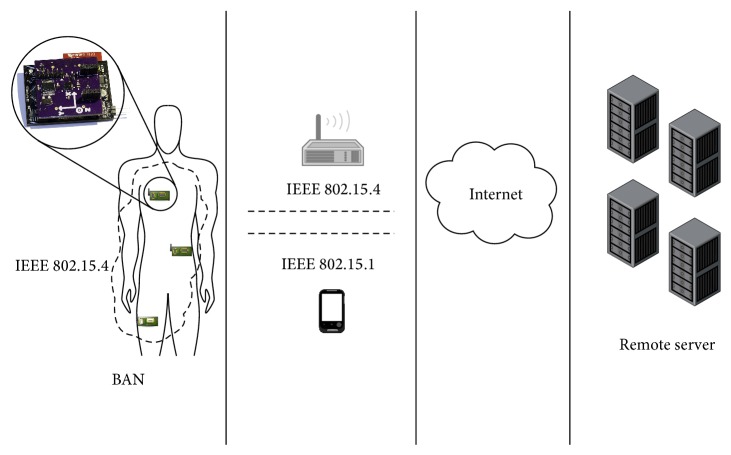
Visual representation of all the parts that make up the physical system architecture.

In our system, there are four main constituents of its architecture: (i) the individual node, (ii) the BAN communication medium, (iii) the remote server (RS), and (iv) the communication medium between the BAN and the RS.  Figure 2 shows a visual representation of all the elements. The system architecture shown in Figure 2 intends to use a technological solution to recognize human movement, identify human postures, and detect harmful activities for preventing risk situations. To achieve these goals, tiny sensors nodes with wireless communication, computational and energy harvesting capabilities are networked around the human body forming a wireless body area network.

The final architecture in which our solution relies is the result of the continuous improvements made to the BodyMonitor system [24]. While it was initially envisioned for implementing a fall detection system, energy constrains had already been taken into consideration being concluded that it was necessary to divide the processing tasks and relay some of them to an external system.

In terms of hardware, we developed our own sensor node so that the platform could be small, lightweight, and very responsive by using data from an accelerometer, gyroscopes, and magnetometers, which enable a more precise reconstruction of human movement [13]. The sensor node is responsible for the acquisition and processing of information relative to the respective body area. It stands as the first layer of decision regarding the importance of the gathered information. In order for the node to be able to apply advanced filters without compromising the remaining activities, it was necessary to use an advanced 32-bit microcontroller instead of a 8-bit one [25]. However, his option involves that during the active phase the power consumption would be higher, leading to lower autonomy. Our solution to this problem was to develop a sensor node which uses ultralow power and a basic microcontroller for the collecting tasks, while the main microcontroller is responsible for carrying out normal system activities and processing the sensor data in batches instead of for every sample. The final microcontroller base board measures 4.8 cm × 3.5 cm × 0.8 cm and weights 11 g while the sensor board measures 3.6 cm × 3.5 cm × 0.4 cm and weighs 5 g.

For intra-WSN communication, the standard that most closely conforms to the project requisites is the IEEE 802.15.6 body area network, which as of June 2014 is still being drafted [26]. As the name infers, the standard was specially designed for communication between devices placed on the human body, being one of the most important aspects its requirement for ultralow power consumption [27]. For the best of our knowledge, as of June 2014 there is no communication device that implements the first draft defined by the IEEE 802.15 task group 6 (BAN) [28]. In such a situation, a good alternative sharing some similarities was the IEEE 802.15.4 (Low-Rate Wireless Personal Area Network) [29]. However, in order to guarantee a possible future transition the constraints defined in the BAN draft were also taken into account.

The RS was designed to complement the BAN. It is responsible for keeping track of the users' information both in terms of individual data as well as common patterns sharable between users. It is also responsible for undertaking heavy processing tasks that would undermine the WSN's energy autonomy such as long term data analyses and agent training, or those that the BAN would not be able to compute in an acceptable time frame.

The system actually supports two forms of communication between the BAN and the RS. The decision on which type of communication should be used depends on the user's location. If the user is inside the home, the communication is done through 802.15.4 enabled wireless access points, thereby minimizing the energy consumption necessary to establish a normal Wi-Fi communication. When out of range, the communication between BAN and RS is done through a smartphone using the Bluetooth radio. The use of this type of communication is more energy efficient and keeps the size of the node reduced by not incorporating an extra GMS radio.

## 4. Multiagent Architecture

With the goal of creating a robust, flexible, and adaptable system, able to take advantage of the capabilities introduced by our node's modern processor, the base principles behind the system architecture shifted from a monolithic design into a multiagent approach. The different parts comprising the final architecture and their relation with the hardware elements are presented in this section.

**Figure 3:**
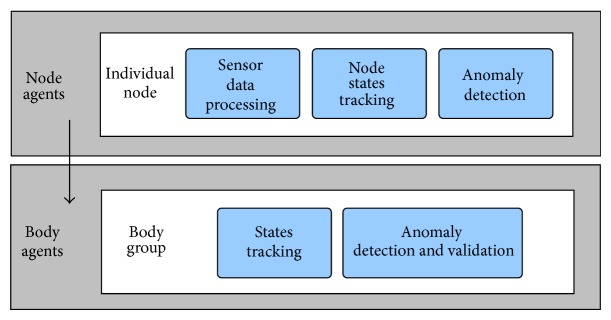
The two layers comprising the proposed multiagent architecture.

The multiagent tasks were devised in two main groups: those related with the management of the individual nodes and those regarding the supervision of the BAN itself. For its part, the BAN architecture was also divided in two complementary layers (see Figure 3 for an explanatory diagram). In Figure 3, the top layer contains multiple groups of agents, each group being responsible for an individual node. Each group of agents is in charge of keeping track of the movement state, orientation states, and preselecting anomalies from the body part in which the node was placed. This information is then passed to the lower layer containing an individual agent group, which is responsible for keeping track of the states, this time in terms of full body movement and orientation. Moreover, the bottom layer is also responsible for verifying if the anomaly detected by a single node could be confirmed or refuted using the data provided by the BAN as a whole.

In our previous architecture, there was already information being exchanged between the nodes in order to correctly evaluate their own data and the state of the whole BAN. Therefore, this could already be considered a primitive type of MAS. However, all the node's processing was focused on data immediately available and the functions used were represented in the format *S* → *A*. In intelligent-agent theory, this conceptualization is defined as purely reactive agents [30].

In this iteration of the work the initial MAS evolved to support a more specific architecture, enabling the system to make decisions based not only in current data but analyzing the event as a whole taking also into consideration previous changes.

The main processing cycle of each wireless node is divided in three blocks:* preprocessing*,* state evaluation*, and* anomaly detection*. As each block presents different characteristics, they have been implemented using separated agents, allowing for a more individualized definition, training, and evaluation.

In order to minimize deployment costs and to create a straightforward system, the required number of body areas being monitored must be reduced. In this context, we decided that the incremental cost and reduced usability necessary to detect very rare ADLs was not justified. The body parts finally selected were the upper torso, hip area and leg. On the one hand, the upper torso and leg were selected in order to quickly characterize normal orientations (standing, laying, and sitting positions). On the other hand, the hip was chosen due to its stability during movement.

**Figure 4:**
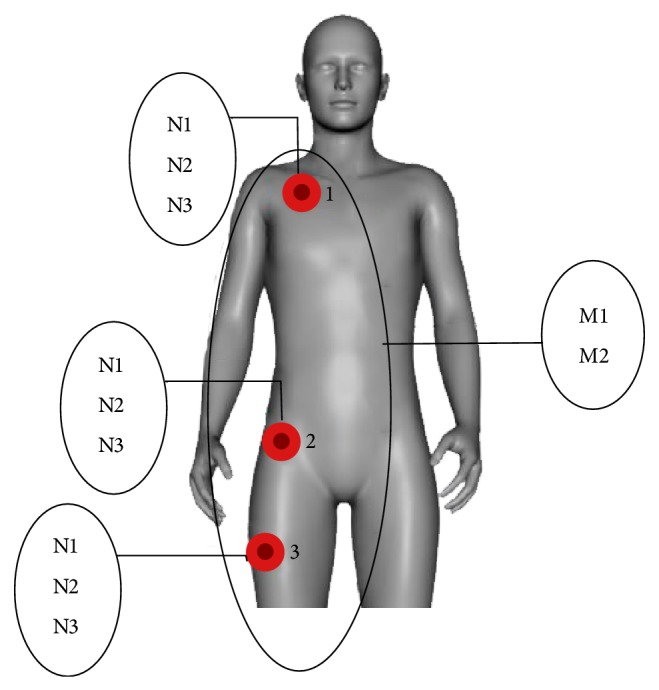
Node placement in different body parts.

Figure 4 shows the relation between node placement and agent groups, in which N1, N2, and N3 refer to the agents responsible for* preprocessing*,* state evaluation,* and* anomaly detection*, respectively, and M1 and M2 stand for the agents responsible for merging the information from the different nodes into full* body states* (M1) and* validating the anomalies* (M2).

## 5. Multiagent Implementation and Deployment

In this section, we provide a detailed discussion about the design, implementation, and deployment of the multiagent architecture presented in the previous section. The framework finally chosen to carry out the agent definition and execution was Jadex [31], who was initially created as an add-on of the widely used JADE platform [32] for giving specific support to the BDI paradigm. Since then, it evolved into an independent middleware with its own unique characteristics, the most notorious being the active component programming model [33] but still keeping complete compatibility with JADE.

The BDI model provides an appropriate way to both conceptualize a system and structure its design by using the concepts of* belief*,* desire*, and* intention* as mental attitudes that generate human actions. Beliefs capture* informational* attitudes, desires* motivational* attitudes, and intentions deliberative attitudes of agents. Rao and Georgeff adopted this flexible representation and transformed it into a formal theory and execution model for software agents based on the notion of beliefs, goals, and plans [34].

In order for each agent to be truly independent, Jadex interagent communication is based on the service oriented architecture (SOA) principles of service searching and binding. This way, the agent does not need to know which other agents will provide it with its required external functionalities, only needing to know which services are wanted and announce them at the same time that publishes those services provided by itself.

**Figure 5:**
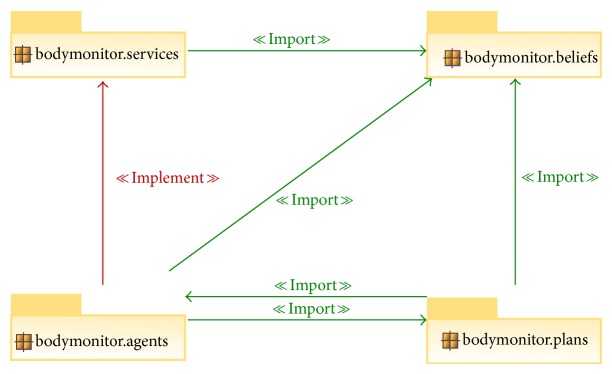
Dependency diagram of beliefs, goals, plans, and services.

With all these features, this framework eases the task of adding new sensors and different complementary functions to our system, as the agent's base stays the same and only the abstract methods should be implemented.  Figure 5 shows the dependency diagram of beliefs, goals (inner class of the agents), plans, and services.

The programmatic representation of the beliefs, goals, plans, and services shown in Figure 5 is as follows.

- Beliefs: they were implemented as classes in order to be easily extended. Agent's beliefs that consist of primitive classes were aggregated in new classes.
- Goals: as they are very specific and share extensive information with its related agent, they were implemented as inner classes. Goals are the implementation of actual desires selected by the agent for active pursuit.
- Plans: given the fact that plans are the part of the agent suffering more changes, they were implemented as independent classes in order to facilitate both subsequent updates and sharing of plans between agents of different types. Plans also stand for the most concrete part of intentions, being constituted by the actions necessary to accomplish a given goal (or a part of it).
- Services: as aforementioned, the communication between agents is done without the source agent having to know the specific implementation of the receiver (target) agent. This is done through the use of Java interfaces, in which the source registers its need for the required service interface and the receiver (or service provider) implements this interface and listens for service requests.

The following subsections are focused on explaining in detail each agent used by our proposed system.

### 5.1. Data Processor (N1): Data Acquisition and Preprocessing

Each Data Processor agent handles the environmental information gathered from the shared memory space. Data is initially transformed from its raw representation according to the conversion table of each sensor, and then, it is filtered using the Madgwick AHRS algorithm [35] to fuse the inertial data into a valid node orientation. This type of algorithm, just like the Kalman filter, is commonly used to minimize the intrinsic error of evaluating a system from the inside. After filtering the sensorial data, and once obtained the fused orientation, the resulting information is passed to the agent responsible for its evaluation (i.e., the State Evaluator agent).

The previous processes are embedded into the BDI reasoning cycle, in which beliefs of the Data Processor agent are continually updated. Those beliefs belonging to the Data Processor agent are defined as follows.

- *Average quaternion*: defined by the stored averaged initial orientations from both the internal (inertial sensors) and the external (computer vision) systems.
- *Fusion filter parameters*: corresponding to the filter configuration data (i.e., the sampling period and, in the case of the Madgwick AHRS filter, the algorithm's gain) together with the internal values of the filter, which are used and updated for each sample.
- *Raw sensor data*: representing the body part raw inertial information acquired by the three inertial sensors.
- *Inertial data*: standardized representation of the sensor data. The accelerometer uses the *g* notation (each *g* corresponds to 9.812865328 m/s^2^), the angular speed from the gyroscope is expressed in rad/s, and the magnetometer magnetic field is denoted in gauss.
- *Fused data*: it represents the orientation resulting from fusing the three sensors and its transformation to the external system's referential. The filtered acceleration is also stored independently.

The Data Processor agent has three main goals.

- *Obtain external reference*: the agent waits and keeps trying to contact an external system in order to get an independent representation of its body part's orientation.
- *Obtain sensor data*: after being awakened by the 8-bit processor, the objective of the sensor is to obtain the inertial data.
- *Have data fused*: once the agent knows the inertial data, the agent's objective becomes its fusion.

In order to accomplish its goals, the Data Processor agent has the following plans.

- *Calculate external referential*: the agent starts collecting and averaging fused orientation data until it receives the equivalent data from the external system.
- *Collect data*: the agent accesses the shared memory and retrieves those samples stored by the 8-bit processor.
- *Process data*: raw data is converted to its standardized representation through each sensor specific conversion table.
- *Fuse data*: data from the three sensors is merged using the Madgwick AHRS filter (loaded using a dynamic class factory). The rotation quaternion which would transform the initial orientation is obtained using *q*_*rot*⁡_ = *q* × *q*_wn_^−1^ (with *q*_*rot*⁡_ being the rotation quaternion, *q* the fused orientation quaternion, and *q*_wn_ the internal system's average orientation). After that, it is applied to the external system's average quaternion using *q*_norm⁡_ = *q*_cv_ × *q*_*rot*⁡_^−1^ (with *q*_norm⁡_ representing the normalized quaternion and *q*_cv_ standing for the computer vision's average quaternion).

**Figure 6:**
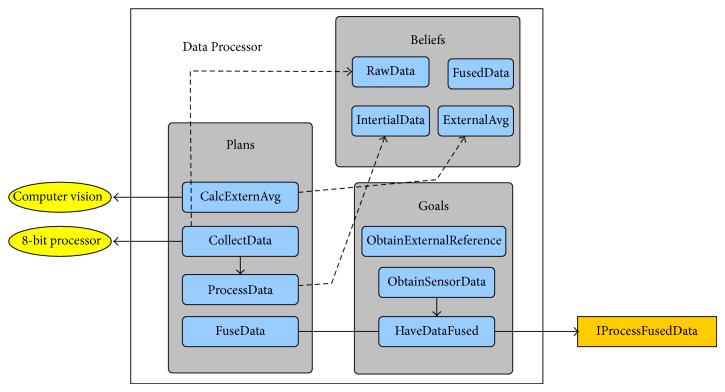
Data Processor agent.

Figure 6 shows a diagram exemplifying the structure of the Data Processor agent.

### 5.2. State Evaluator (N2): Asserting Node Orientation and Movement States

Although the received information from the Data Processor agent is already fused, it is only relative to a single point in time. Therefore, the State Evaluator agent is responsible for transforming this data into a continuous stream of information. The fused data is then reprocessed and complementary information is calculated (i.e., velocity, walking distance, step cadence, and step energy).

After inferring the continuous information, the State Evaluator agent estimates the current state of the node regarding its orientation and verifying whether it is in motion or stationary. In case a change of state is detected, the node responsible for keeping track of the full body state is contacted. The inferred data, together with the states, are then sent to the Anomaly Detector agent. This data is also made available to the agent responsible for carrying out anomaly validation.

As a common BDI agent, the State Evaluator agent has its own beliefs, goals, and plans, also providing services for other agents. The specific beliefs belonging to the State Evaluator agent are defined as follows.

- *Inferred data*: representing data inferred from the data fused by the Data Processor agent. It is composed by velocity, activity, step length, walking distance, step cadence, and step energy.
- *States*: comprising movement states (*moving* and* stationary*) and orientation states (*vertical* and* horizontal*).
- *Personal data*: composed by the physical characteristics of the user as well as his/her normal orientation and movement ratios.

The State Evaluator agent has two main goals.

- *Obtain inferred data*: once received the fused data, the goal of the agent becomes to infer extra information from it.
- *Obtain node state*: for keeping the track concerning the state of each node. This goal is further divided into two subgoals: (i) obtaining the movement state and (ii) obtaining the node's orientation state. The general goal is accomplished when both subgoals are achieved.

In order to accomplish its goals, the State Evaluator agent possesses the following plans.

- *Infer data*: in this plan, the fused data is processed for obtaining the inferred data. In particular, the accelerometer's proper acceleration must be converted into dynamic acceleration using the formula ${\vec{a}}_{\text{dyn}}={\vec{a}}_{\text{prop}}-\vec{g}$ (for a better understanding of how the gravity's direction is obtained refer to [36]) and then integrated into velocity using the formula $\vec{v}\left(t\right)=\int {\vec{a}}_{\text{dyn}}(t)dt$. For activity detection it is used the Acceleration Moving Variance Detector (AMVD) function defined in [37], with the formula being $\left(1/N\right)\sum_{k=1}^{N}{\left\|a_{n}-{\overset{-}{a}}_{k}\right\|}^{2}<\gamma$ where *a* stands for acceleration,  *γ* is the threshold (in this case 0.0013), and *N* represents the window size (in this prototype the size is 20). If the agent corresponds to the hip node, the step detection is done using the acceleration peak-to-peak variation, the step cadence is calculated by integrating the velocity computed for the duration of the step, the walking distance is calculated by adding the multiple step distances, and step cadence is obtained by averaging the number of steps in each sample during the walking activity. If the agent corresponds to the leg node, the step force is also calculated by multiplying the user's weight by the acceleration when the step impacts the ground (step's acceleration peak).
- *Assert node movement state*: for each sample, the movement of the nodes is evaluated based on the sample's calculated activity (i.e., the state is set to moving if the threshold is passed in the AMVD function).
- *Assert node orientation state*: the possible states of the node's orientation are horizontal or vertical, and for each node and state there is a corresponding transition interval in the user's profile. For each sample the orientation is checked against the current state's transition interval, and if the orientation value lies within that same interval the state is changed, otherwise the state remains the same.

Moreover, the State Evaluator agent provides to other agents the following services.

- *Process fused data*: it provides the capability of processing fused data. This service triggers the* Obtain inferred data* goal.
- *Supply inferred data*: other agents may request individual samples (or complete sampling periods) of data already inferred.

**Figure 7:**
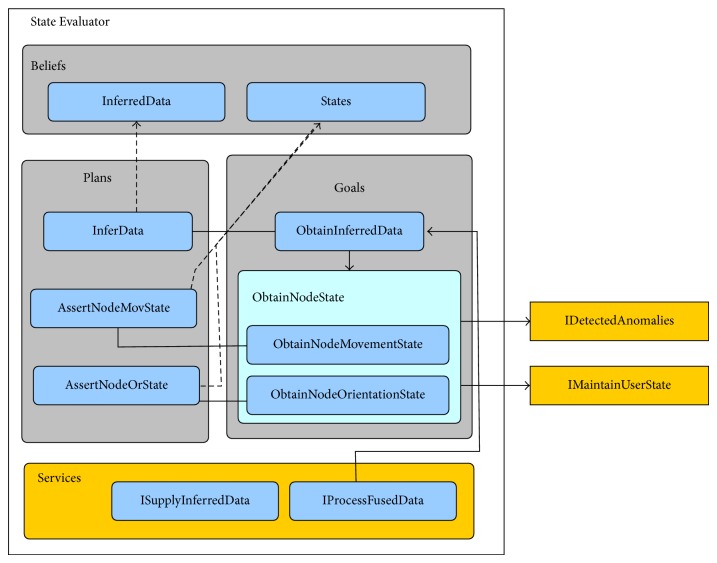
State evaluator agent.

Figure 7 shows a diagram exemplifying the structure of the State Evaluator agent.

### 5.3. Anomaly Detector (N3)

This agent is responsible for ascertaining whether the data coming from the State Evaluator agent is normal or it presents any kind of anomaly. The type of anomalies being investigated depends on the position of the nodes, but all of them are searched looking for sudden variations in the inferred data that could have caused an accident. Due to the fact that each node only maintains information belonging to its own position, the degree of filtering done to avoid false positive errors is very small. Therefore, any suspicious deviation is sent to the node responsible for verifying full body anomalies.

The specific belief of the Anomaly Detector agent is the following.

- *Anomaly history*: composed by the log of all possible anomalies detected by this node.

The Anomaly Detector agent has one specific goal.

- *Have data checked*: triggered by the* Detect anomalies* service. Tries to detect if any of the samples contains suspicious information.

In order to accomplish its goal, the Anomaly Detector agent has one plan.

- *Verify inferred data*: fused and inferred data belonging to each sample is compared to the user's range of normal values stored in its profile. To do so, the agent only has to verify if the detected value lies within the interval defined in the user's profile. This interval is defined and updated in the RS and already includes the necessary threshold tolerances (i.e., the tolerance is dependent on the deviation of values in the aforementioned interval and is different for each individual user and each profile parameter) so that the BAN node can avoid extra processing. Each sample that strays from the predefined range is added to the anomaly log.

Moreover, the Anomaly Detector agent provides to other agents the following service.

- *Detect anomalies*: point of communication enabling the evaluation of inferred data from other agents.

**Figure 8:**
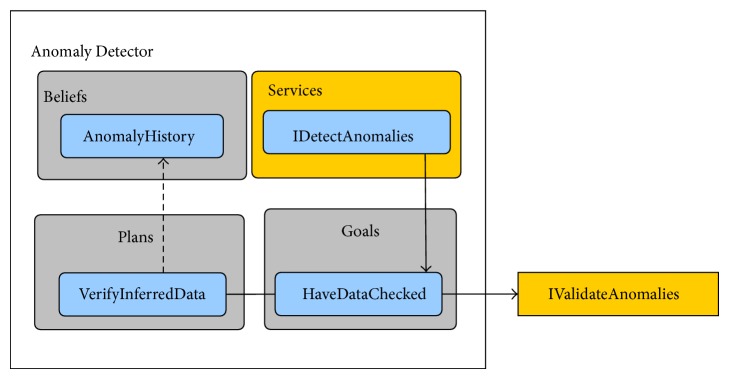
Anomaly Detector agent.

Figure 8 shows the corresponding diagram to exemplify the structure of the Anomaly Detector ECTOR agent.

### 5.4. Body State Evaluator (M1)

Body State Evaluator is the first mobile agent of the main agent group, being purely reactive. The main function of this agent is to keep updated the state machine which maintains the user movement and orientation as a whole. For this purpose, the Body State Evaluator agent uses information already processed by other State Evaluator agents.

This agent possesses a migratory capability and it is able to visit a set of nodes while maintaining its present information state. The specific belief of the Body State Evaluator agent is the following.

- *States*: comprising both the individual change in the state of each node state and the movement and orientation states from the full body itself. Full body movement states are* moving* and* stationary* while orientation states are* standing*,* lying,* and* seated*.

As in the previous case, the Body State Evaluator agent has one specific goal.

- *Obtain node state*: this goal is comprised of two subgoals: obtaining (i) the full body's movement state and (ii) the full body's orientation state. The general goal is accomplished when both subgoals are achieved.

In order to accomplish its goal, the Body State Evaluator agent possesses the following plans.

- *Assert movement state*: as the movement state is only based on the hip node, whenever it received a movement state change from this node the plan automatically defines the full body movement state as the same as the one just received.
- *Assert orientation state*: the decision of changing the current orientation is done based on the data received from individual nodes. As an example, when the leg node informs that it is in horizontal state, if the last orientation communicated by the chest's node was horizontal then the full body's current state is laying, otherwise is sited.

Moreover, the Body State Evaluator agent provides to other agents the following services.

- *Supply full body state*: on request, this agent provides other agents with the correct information concerning the states.
- *Maintain user state*: receives information from the states of the individual nodes with the goal of maintaining the full body state.

**Figure 9:**
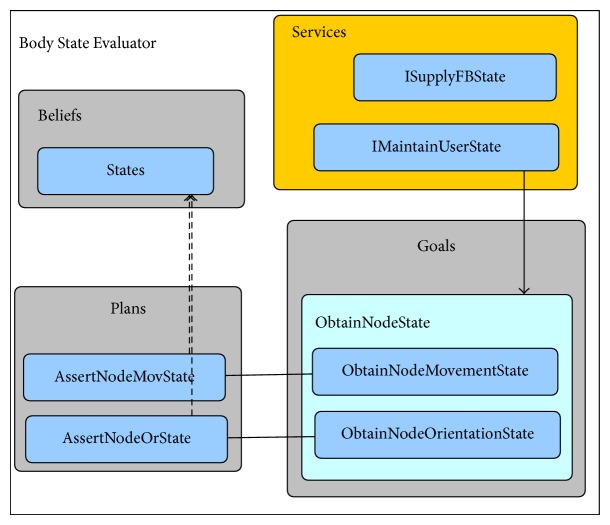
Body state evaluator agent.

Figure 9 shows a diagram exemplifying the structure of the Body State Evaluator agent.

### 5.5. Anomaly Validator (M2)

This mobile agent acts as a final step between all the detection work done inside the BAN and the remote server. Each time a node processes information suspicious of representing an anomaly, this agent is contacted.

The first stage of the validation process consists on collecting information from other nodes and consulting the Body State Evaluator agent regarding the current orientation and movement states of the user. Afterwards, data coming from the three body parts is compared. If a conclusion is taken, the server is immediately contacted or the rejected anomaly is simply logged. Alternatively, if the result is inconclusive, the agent waits for another sampling period. In order to make a decision in the absence of a definite result, the server is contacted and only just data is sent to it. As an example, in the majority of the hampered falls no node will return an impact that will cause (by itself) an immediate alert, but if the following samples show no user movement, the server is contacted to guarantee that no fall is missed.

The specific belief of the Anomaly Validator agent is the following.

- *Confirmed anomalies*: containing a list of anomalies that were confirmed by this agent.

As in the previous case, the Anomaly Validator agent has one specific goal.

- *Have anomalies validated*: the only objective of this agent is to verify if all the possible anomalies detected by individual body part nodes are actually true anomalies.

In order to accomplish its goal, the Anomaly Validator agent has one plan.

- *Verify anomaly*: the agent contacts other agents in order to verify if the data belonging to the anomaly verification request is actually a true anomaly or just a rare data occurrence. The request-and-evaluate cycle continues until a decision can be taken.

Moreover, the Anomaly Validator agent provides to other agents the following service.

- *Validate anomalies*: service that enables those nodes belonging to different body parts to process their anomalies using full body information.

**Figure 10:**
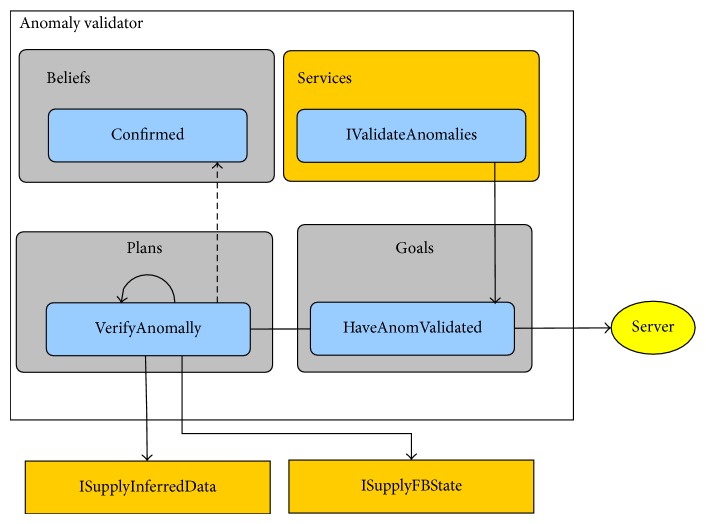
Anomaly validator agent.

Figure 10 shows the corresponding diagram to exemplify the structure of the Anomaly Validator gent.

### 5.6. Agent's Communication

In order to facilitate the comprehension of our system, this section summarizes the communication process carried out by all the agents comprising the proposed approach.

In summary, the data analysis cycle starts when the Data Processor agent is awaken by the external processor, which promptly proceeds to filter and fuse available signals. The resulting data is then sent to the State Evaluator agent, so that extra information can be extracted from the orientation and acceleration data. At this point, it is also verified if the movement and orientation states have changed. If it is the case, the Body State Evaluator agent is informed in order to track the full body state in a central location. Both the inferred information and the fused data are then aggregated and sent to the Anomaly Detector agent for testing the presence of anomalous data. If such data is found, then the Anomaly Validator agent is contacted. With the goal of correctly verifying if the data actually represents an anomaly, this agent may request extra information from both the remaining State Evaluator agents and the Bogy State Evaluator agent. In case an anomaly is actually detected, the remote server is subsequently notified.

**Figure 11:**
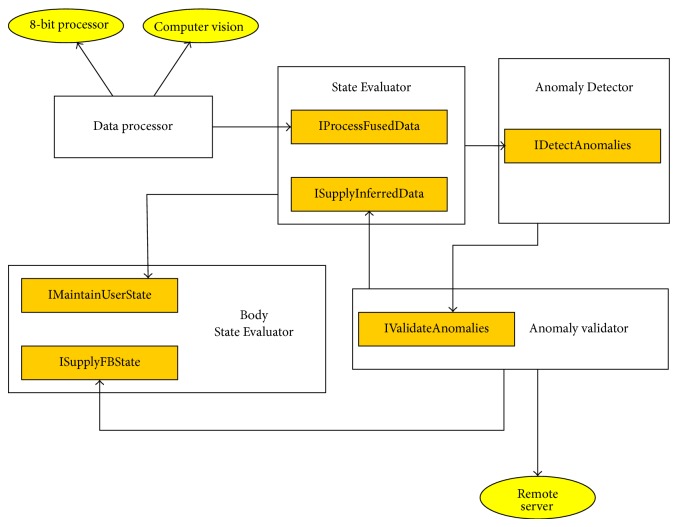
Integrative communication diagram.

Figure 11 introduces the complete communication diagram with the goal of illustrating the interaction inside the multiagent system and between the MAS and the external devices.

## 6. Results and Discussion

To assert the validity of our proposal, two different scenarios were conceived. The first one consists of tests specifically designed to verify the correct movement recognition and accident detection capabilities of our system. The second one was defined taking into account the inner working of the multiagent system, together with the objectives behind the migration to an agent-based platform. This section first describes both case studies and latter discusses the results.

### 6.1. Case Study Number 1: System Accuracy for Movement Monitoring and Accident Detection

While the algorithms in use were already tested in our previous research, it was still mandatory to corroborate if they were correctly implemented in their respective agents and how they perform.

The following experiments were repeated ten times by each volunteer of the testing group. The group consisted of six members (three female and three male) being their average height and weight 173.50 cm and 75.33 kg, respectively, with a standard deviation of 5.02 and 11.18. The average age was of 27.33 years with a standard deviation of 3.64. All the volunteers participating in this study gave their informed permission for the use of the data collected during the proofs.

Movement recognition tests were conducted in two phases. The first one acted as a control stage with all the actions being conducted without mishaps. During the second phase, the volunteers were asked to simulate a problem associated with the specific movement being studied. In particular, the movement recognition experiment tested the following situations.

- *Standing*: the control phase consists of standing straight and immobile for ten seconds. In the second stage the volunteer was asked to move the body without leaving the initial position, again for ten seconds. The objective of this try is to verify if the MAS is able to correctly differentiate between a body part movement and locomotion.
- *Walking*: in the control phase the volunteer was asked to walk in an “L” shaped pattern, stopping in the end of each sequence before continuing. In the problem simulation stage, the volunteer was asked to carry out one of the following actions: (i) simulate a small hump while walking or (ii) walk with a very different stepping cadence from the one used in the control phase. Both subcases were repeated 10 times individually.
- *Sitting*: in the control phase the volunteer sited down correctly (with his back strait), while in the second stage he carried out this activity with an excessive longitudinal or lateral inclination.

Regarding accident detection evaluation, the objective was to verify (i) if unhampered falls continued to be correctly detected and (ii) if by using our agent-based system it was possible to obtain a better ADL differentiation inside the BAN. The fall tests consisted of the volunteer falling into a mattress both from a stationary position and while initially walking. As the ADL responsible for triggering false fall events is rough sit-down actions, the volunteer was asked to sit down by letting his body drop into a chair instead of completely bending his knees.

In terms of hampered falls (i.e., those with an initial deceleration before the impact with the ground), it was also tested if the added internal data evaluation allowed for a higher detection rate. The experiment involved asking the volunteer to initially impact with a vertical mattress and then falling to a mattress placed at ground level, being immobile after the final impact.

### 6.2. Case Study Number 2: Multiagent Performance

There are several reasons why it was selected the Jadex framework for migrating the existing architecture to a MAS approach: its modularity, the ability to easily introduce new agents, and the facility to include new system capabilities and/or change/upgrade existing ones. However, in order to guarantee that core functionalities of the base system are correctly implemented and object-oriented programming rules are fully respected, we used the CKJM extended [38] testing tool to calculate Chidamber and Kemerer object-oriented metrics.  Table 1 shows a summary briefly describing the purpose of each selected metric.

To the best of our knowledge, there is no MAS created with the same objectives as ours, so there are no defined metrics to compare to. Nevertheless, in order to be possible to evaluate future iterations of this project and also to allow that different projects can compare their results with ours, we further computed and collected the following parameters.

- Number of false state changes but corrected at least in the next sample. This situation implies that there is still an error in the state analyses, but a very low value might also mean a very low sensibility, where actual states changes might be ignored.
- Number of internal false anomalies but correctly verified by the Anomaly Validator. Even if anomalies are correctly verified, extra energy was spent for intra-BAN communication.
- Number of internal false anomalies during an ADL but correctly verified by Anomaly Validator. Some ADLs tend to be confused with accidents, so an adequate balance must be found for guaranteeing that any accident is ignored and, at the same time, the number of unnecessary communications does not impair system autonomy.
- Number of internal false anomalies while resting but corrected by Anomaly Validator. In this case, even a moderate value implies that node agents are correctly executing the anomaly filtering task.
- Number of different events triggered during an accident. It is important to understand how the node reacts during an accident and also how a high/low value impacts the algorithm performance.

### 6.3. Results

We will start by describing the results from case study number 1, more specifically the tests concerning the movement recognition component of the agents. These results are presented in Table 2.

As expected, results shown in Table 1 do not present any statistical difference from those obtained by our previous system, as the changes done to this component of the architecture were related to efficiency and not accuracy. It is important to notice that in the case of walking with a small hump, what was being tested was the distortion in the walking pattern, so the alerts of the anomalous inclination were only considered during the actual walking phase, otherwise the accuracy would have been 100%.

The second part of the algorithm evaluation tests consisted on studying their efficiency regarding fall detection. The results from these experiments are shown in Table 3.

In terms of unhampered fall detection, the conducted tests did not present results statistically different from the ones belonging to our previous system, being all the falls correctly detected.

Regarding the detection of unhampered falls and its differentiation from ADLs, the new algorithm revealed a clear increase in accuracy (83.33% against the precious 59%). This increment is due to both the analysis of more parameters (not only the acceleration but also the final full body state of the users) and the analysis of multiple sources of data (the sudden deceleration from one node may not be high enough to trigger an alert but two or more high values from different sources are).

**Figure 12:**
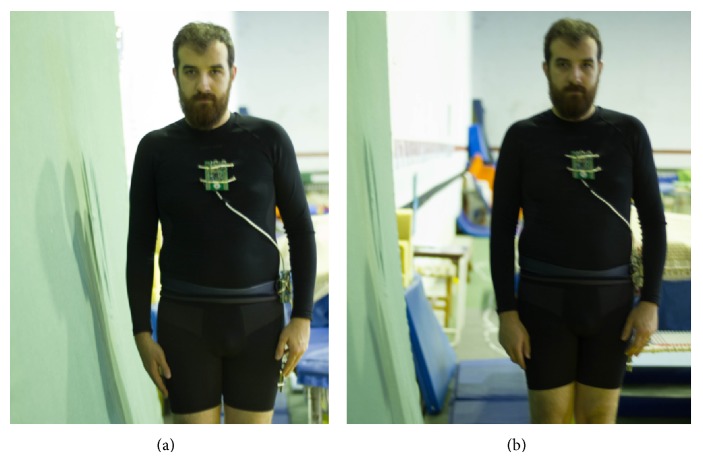
Demonstration of the distance to the initial contact. In (a) the distance is not enough for the impact to cause harm or to trigger an alert; in (b) the distance is enough to cause harm and to trigger the alert.

After interpreting the data from the not detected falls, it was concluded that it was due to one of the two following reasons. The primary cause, and the one that was not possible to smooth out of the algorithm, is related to the falls where the distance between the beginning of the movement and the impact is so small it would not even harm the user (visually explained in Figure 12). The problem of these falls not being detected is not relative to the injury of the fall itself, but the fact that they might imply another type of health problem. If the algorithm is modified to encompass these types of falls, even the most basic ADL may trigger an alert.

**Figure 13:**
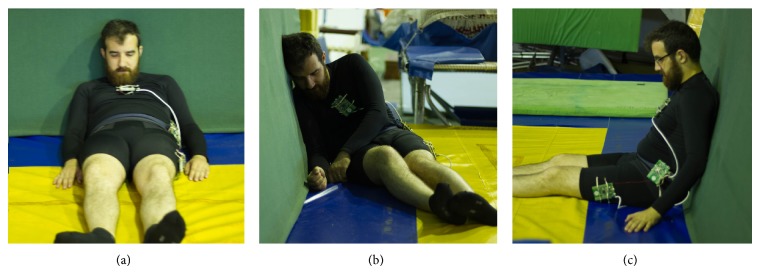
Examples of how the body stays after a hampered fall. In (a) the body fully slid of the wall ending in a laying position; in (b) the user ended in a sitting position but his posture is incorrect; in (c) the user slid to a sitting position fully supported by the wall.

The second cause for misclassifications is related to the similarities between a hampered fall and the sit-down action. This type of falls stays undetected when the sudden decelerations of the multiple impacts are similar to the decelerations of a rougher sit-down action. In a first testing stage, part of this limitation was corrected by taking into account the user's final orientation state. In this line, if the user was lying down or sitting, but in an incorrect sit-down position, the alert is sent. In the event the acceleration is very similar to a rougher sit-down action, and the user final orientation is consistent with a normal sitting position, the fall stays undetected (although the information is still sent to the remote server).  Figure 13 shows some examples of the aforementioned after fall positions.

With reference to the results from case study number 2, the outcomes belonging to the Chidamber and Kemerer metrics are presented in Table 4.

The very high standard deviation of the majority of the metrics is due to the big different between the more supporting classes (utility classes, abstract classes, or even, the beliefs and plan classes) and the actual agent classes. The agents were the only classes with high WMC, coupling, or RFC values, the remaining classes presented very low values, which helps to demonstrate the great scalability of the proposed system.

Finally, with respect to the tests concerning the proper operation of our system, Table 5 shows the obtained values. Results were very satisfactory, with the number of unnecessary node-to-node and node-to-server communications being kept low. However, there were some situations of unnecessary communication present in the system, as will be discussed below.

In regard to incorrect state transitions (i) the number is low, being mainly related to the transition between active and inactive periods primarily during slow walks, and very rarely lead to node-to-node communication. In the case of the number of false anomalies (ii, iii), the great majority is related to the misclassification of body orientations during posture transitions. As these anomalies are not maintained for more than a few samples, there is no node-to-node communication. Regarding anomalies which may imply accidents (iii), as expected the only ADL that triggered this type of anomaly was the rougher sit-down action. In terms of false anomalies while stationary (iv) there were none detected. The normal events that took place during an accident were full body activity change, full body orientation change, and high acceleration alert. The number of individual alerts was conditioned by the final body orientation and the force of the individual impacts.

## 7. Conclusions

In this paper, a multiagent system for recognizing human movements, identifying human postures, and detecting harmful activities is presented. Using the proposed multiagent architecture, we have focused on how to fuse information from different agents which belong to the same objective, allowing them to correct errors by their ability to make an explicit coordination.

Experimental results on two different scenarios demonstrate that the designed architecture can successfully evaluate the user's movement and posture. The accuracy achieved enables a long term study and the detection of both beneficial and harmful changes. The performance finally obtained also represents an improvement over our previous system with respect to hampered fall detection. This gain was only possible by enabling individual nodes to work as a group instead as a set of isolated individuals.

In addition to these results, the use of a multiagent architecture for a BAN applied to healthcare monitoring brings several advantages with respect to traditional systems. In this context, one of the primary benefits is the scalability of the whole system, since it could be possible to increase the monitoring area by adding more agents without decreasing the performance of each agent. Complementarily, the use of agent-based standard communication protocols enables the system to achieve a higher abstraction level of interoperation with other systems.

Mobile agents, with migratory capabilities for visiting several nodes, are able to accumulate information corresponding to the user movement and orientation as a whole. Benefits include low overall computational costs, as no more than one node is active at the same time instant. Since a single transmission and reception event is required from an individual node to facilitate the agent's migration, communication is routinely low. Moreover, simplicity is another important advantage, since the agent manages both the task of collecting data and its visitation route.

Future work will consider the introduction of new machine learning algorithms into the Anomaly Detector and Anomaly Validator agents in order to more precisely detect hampered falls. The goal is to be able to detect those falls whose difficulty comes not from the fall itself, but from other health conditions (e.g., stroke, seizure, and/or fainting). We are also interested in improving the remote server for better exploiting the advantages brought by the proposed MAS, enhancing its capacity to carry out long term evaluation of the user's condition. Finally, we intend to run long term tests of the new architecture alongside the previous one in order to better evaluate the practical benefits of the final stage of the new architecture.

## Figures and Tables

**Table 1:** Chidamber and Kemerer metrics analyzed in our system.

Metric	Description
Weighted methods per class (WMC)	Measures the complexity of the each class based on the sum of the cyclomatic complexity of each of class's methods.

Depth of inheritance tree (DIT)	Measures the inheritance level of each class. A high DIT number means class extension is more prone to error as each intermediate class introduces new methods and variables whose access must be controlled. Faults also become harder to detect.

Number of children (NOC)	It counts the number of immediate child classes each class has. A class with a high NOC is very hard to change (or update) due to the number of classes reusing it.

Coupling between object classes (CBO)	Measures the relationship between classes and how dependent they are of each other. A high coupling rate is unwanted in a very modular system such as the presented in this work. Additionally, two submetrics of CBO can be also computed: *afferent couplings* (Ca) that measures the number of classes that use each class and *efferent couplings* (Ce) that indicates the number of other classes being used by each class.

Response for a class (RFC)	Stands for the potential number of methods that can be called when one of the class's methods is invoked. In this test, a special importance is given to remote methods, as they not only increase the complexity of the class but also augment class coupling.

**Table 2:** Result of the movement recognition tests.

Group	Test	Accuracy
Standing	Control	100%
	Problem	98.33%

Walking	Control	100%
	Problem (hump)	81.67%
	Problem (step cadence)	100%

Sitting	Control	100%
	Problem	90%

**Table 3:** Result of the fall tests.

Fall type	Accuracy
Unhampered	100%
Hampered	83.33%

**Table 4:** Chidamber and Kemerer metrics for the proposed system.

Metric	Average	Std. deviation	Min	Max
WMC	8.72	7.19	1	28
DIT	0.83	0.47	0	2
NOC	0.17	0.62	0	3
CBO	5.73	5.49	0	29
Ca	1.98	2.41	0	9
Ce	3.79	5.33	0	27
RFC	13.79	11.45	1	64

**Table 5:** Results of movement recognition tests.

Test	Summary	Result
i	False state changes	0.67
ii	Internal false anomalies	0.11
iii	False anomalies during an ADL	0.06
iv	False anomalies while resting	0.00
v	Events per accident	0.33
